# Explaining the barriers faced by veterinarians against preventing antimicrobial resistance: an innovative interdisciplinary qualitative study

**DOI:** 10.1186/s12879-024-09352-7

**Published:** 2024-04-30

**Authors:** Razie Toghroli, Laleh Hassani, Teamur Aghamolaei, Manoj Sharma, Hamid Sharifi, Maziar Jajarmi

**Affiliations:** 1 Social Determinants in Health Promotion Research Center, Hormozgan Health Institute, Hormozgan University of Medical Sciences, Bandar Abbas, Iran; 2 Department of Social and Behavioral Health, School of Public Health, University of Nevada, Las Vegas (UNLV), Las Vegas, NV 89119 USA; 3 HIV/STI Surveillance Research Center, and WHO Collaborating Center for HIV Surveillance, Institute for Futures Studies in Health, Kerman University of Medical Sciences, Kerman, Iran; 4 Department of Pathobiology, Faculty of Veterinary Medicine, Shahid Bahonar University of Kerman, Kerman, Iran; 5 Institute for Global Health Sciences, University of California, San Francisco, CA, USA

**Keywords:** Antimicrobial resistance (AMR), Food Safety, Veterinarian, Qualitative research

## Abstract

**Background:**

Considering the significance of increased antimicrobial resistance (AMR) and its adverse effects on individual and social health and the important and effective role that veterinarians play in controlling this growing issue worldwide, it is essential to have effective preventive control programs. To this aim, the first step is to identify the factors behind the prevalence of AMR in Iran and the barriers veterinarians face to controlling this problem. Thus, the present study was conducted to explain the barriers veterinarians faced in the prevention of AMR from an Iranian veterinarian’s perspective.

**Methods:**

The present research was done in three cities in Iran in 2021. The data were collected through in-depth interviews with 18 veterinarians selected through purposive and snowball sampling and analyzed using conventional qualitative content analysis.

**Results:**

The data analysis results were classified into 4 main categories and 44 subcategories. The former included: educational factors, administrative/legal factors, client-related factors, and veterinarian-related factors.

**Conclusions:**

The increased AMR can be approached from multiple aspects. Considering the different factors that affect the increased AMR, it is necessary to consider them all through effective planning and policy-making at multi-level and multidisciplinary dimensions. There is special attention needed to scientific and practical interventions at the individual, interpersonal, social, and even political levels. At the same time, measures should be taken to rehabilitate and maintain the health of society to strengthen supervision and attract the full participation of interested organizations.

## Introduction

Antimicrobial resistance (AMR) refers to the reduced effectiveness of antimicrobial agents, such as antibiotics, antivirals, antifungals, and antiparasitics, against infections caused by bacteria, viruses, fungi, and parasites [1]. This phenomenon makes infections harder to treat and increases the risk of disease spread, severe illness, and death. Misuse and overuse of these agents in humans, animals, and plants are key contributors to the development of AMR. AMR is a natural process that occurs gradually over time through genetic changes in microorganisms, but human activities, particularly the improper use of antimicrobials, significantly speed up this process. Veterinarians play a vital role in managing AMR, as they frequently prescribe antimicrobials to protect animal health. However, overprescription and misuse of antimicrobials in veterinary practice contribute to the development of AMR in humans. AMR presents a substantial challenge to global public health and economic stability. If left unchecked, AMR will lead to increased healthcare costs, decreased productivity, and potentially millions of avoidable deaths annually. To combat AMR, governments, healthcare providers, and researchers must collaborate to implement policies promoting judicious antimicrobial use, invest in innovative therapies, and foster educational initiatives to empower individuals to understand the importance of responsible antimicrobial stewardship [2].

AMR is an increasingly global issue that needs to be settled cooperatively. Resistant organisms exist in animals, humans, the environment, the food, and the main cause of this, is antimicrobial usage. AMR will become a leading cause of mortality in the world in the near future. As reported by some studies, by 2050, AMR will be the main cause of death on a global scale, which surpasses cancer deaths [3, 4].

The mortality rate caused by microbial resistance is higher than the total number of deaths induced by cancer worldwide. Yet, the former has been largely neglected and, instead, issues such as cancer and how to treat it have been addressed more [5–7].

Unintentional antibiotic ingestion occurs frequently due to the widespread use of antibiotics in society. According to a report from 2010, approximately 10 pills, capsules, or teaspoons of antibiotics are taken annually by every person on Earth, which suggests a high degree of accidental consumption. Healthy individuals who consume significant amounts of antibiotics unintentionally may experience negative impacts on their health, particularly concerning the disturbance of the normal microbiome. This disturbance can lead to long-term complications, such as an increased risk of developing conditions like type 1 and 2 diabetes, inflammatory bowel diseases, celiac disease, allergies, and asthma [8]. Additionally, antibiotic exposure can contribute to the emergence of antibiotic-resistant strains, posing a challenge to public health. It is essential to note that antibiotics are necessary and lifesaving medicines when used appropriately under medical supervision. However, excessive or unnecessary use of antibiotics can pose risks to individual and population health [9, 10].

According to the report of the World Health Organization, half of the antibiotics produced in the world are used in medicine and the other half in veterinary, agriculture and aquaculture [11]. In general, there is no difference between antibiotics used in veterinary medicine and antibiotics used in medicine. These drugs are used to prevent and treat diseases and promote growth in animal farms (pigs and poultry), unfortunately, the use of antibiotics in veterinary medicine leads to leaving residues in meat, milk and eggs [12]. Drug residues in food have adverse effects such as antibiotic resistance in humans, allergies, and inhibition of bacterial starter cultures used in dairy fermentation industries [13]. Despite the beneficial effects of antibiotics on the treatment of livestock infectious diseases, the presence of their residues in milk and animal meat, as well as their transfer to the human body have adverse effects on health, industry, and economics. As reported by the National Center for Rational Prescription of Antibiotics, consuming antibiotics in Iran is 16 times as high as the global standard. Some researchers believe that the spread of microbial resistance to antibiotics results not only from the unnecessary prescription and use of these compounds in humans but also from the widespread use of antimicrobial drugs in veterinary medicine. It has caused the transfer of such pathogenic bacteria from animals to human pathogens. The main difference between microbial resistance to antimicrobial drugs in humans and animals is that microbial resistance in humans affects the individual, whereas microbial resistance in livestock affects a large population due to the consumption of raw animal products by humans. Exposure to both resistant bacteria and antibiotic compounds prescribed for the treatment of infectious diseases for livestock through transmission causes the accumulation of drugs and drug residues in raw livestock products. It seems that attempts to prevent the occurrence of microbial resistance in livestock and its consequences for humans are effective and can be implemented efficiently by veterinarians and those active in this domain. What veterinarians can do with this respect is wide-ranging.

Most of the studies conducted in Iran in the field of antimicrobial resistance were in the medical and human fields, and the studies conducted in the veterinary field were mostly quantitative. A systematic review and meta-analysis showed a high level of antibiotic resistance in Staphylococcus aureus bovine mastitis in Iran. This pathogen is the common and main cause of bovine bacterial mastitis, which leads to high economic losses and can easily lead to the transmission of these treatment-resistant bacteria to humans [14]. In a qualitative study, which is one of the few qualitative and phenomenological studies conducted in Iran in the field of AMR, the lived experience of livestock breeders, their role and views in this field has been investigated. The results of this study have confirmed the importance of antibiotic resistance in Iran and the lack of existing research in this field, especially with a qualitative approach [15]. In another study that was conducted with semi-structured interviews with key stakeholders in Iran, including managers of the Ministry of Health, Iran Veterinary Organization, national professional associations and researchers through thematic analysis, the international enabling and predisposing factors related to It identified the control of AMR in Iran. The enabling factors that have been highlighted in this review were discussed in general, and more attention was paid to political factors such as formulation and implementation processes, and AMR surveillance, and challenges such as the smuggling of infected animals and antimicrobial drugs and livestock from neighboring countries and the impact of imposed sanctions. The review emphasizes the global nature of AMR as a challenge that requires consensus and international cooperation to effectively deal with this issue, but it does not specifically and specifically deal with why and analyze how AMR occurs and examine ways to prevent it, and only generally with the approaches Emphasizes political, including health diplomacy, to strengthen national efforts in the fight against AMR [16]. However, the present study, in an interdisciplinary manner, has specifically addressed one of the most important fields involved in the occurrence of AMR in human societies, and a field similar to it has received less attention before.

Yet, it is hard to make interventions with veterinarians directly involved because they are not easily available for research; therefore, veterinary students are the best and closest population for interventional studies. If this population adequately understand the principles of prescribing antibiotics, this successful learning will be productive in practice too [17].

Overcoming this problem will be possible with an One health approach, taking into account humans, animals, and environmental health altogether [18–20].

Today, AMR occurs in humans, wildlife, domestic animals, plants, and our environment directly by using antibiotics, and there is a risk on a much larger and more significant scale in animal-source foods consumed by humans indirectly. So it is logical to take a multidisciplinary health approach to solve this problem by eliminating the inappropriate use of antibiotics [21, 22].

In the medical domain, extensive research has been done to examine physicians’ beliefs about prescribing antibiotics [23]. Many interventions have been made to reduce physicians’ over-prescription. The various aspects of antibiotic prescription have been extensively investigated so far in medical and clinical sciences. However, these interventions alone have not managed to prevent the occurrence of this important event. Thus, resolving this problem needs a multidimensional approach [24] .

Moreover, veterinarians prescribing drugs without using paraclinical services and selling over-the-counter (OTC) drugs are very common in several countries including Iran. In many stockbreeding industries, antibiotics are widely used not only for medical purposes but also as growth stimulants. There has been a serious lack of effective monitoring of these patterns of use. Similarly, there has scarcely been any strict preventive rule for this. Thus, it is likely that the AMR incidence rate is high in countries such as Iran [25, 26].

A vast majority of research so far on the effect of AMR has only addressed this issue in human health [16, 24]. In veterinary medicine, the body of existing literature has been limited to laboratory research and animal health. Veterinarians’ role in integrated health, especially AMR has not been adequately addressed. Therefore, there is no complete and clear understanding of veterinarians’ mental patterns and perceived social barriers to their decisions during diagnosis and treatment [27]. 

The over-prescribing of drugs is very common in animal products and animal-source foods (to be consumed by humans) in Iran. Moreover, each of these foods contains antimicrobial residues. Thus, it can be conjectured that people ingest significant amounts of antibiotics every day unintentionally without suffering from any infectious disease. Therefore, veterinarians must pay adequate attention to AMR in human health [28].

Overall, the world is faced with increasing availability and misuse of antibiotics in veterinary medicine, which threatens public health. There is a significant increase in AMR on a global scale, and there is a threat of increasing infections that do not respond well to treatment. It is essential to take appropriate measures and plan to prevent the over-prescription of antibiotics by veterinarians. There is an increasing need for education and empowerment policies, all deemed impossible unless the barriers facing veterinarians are recognized in appropriate prescribing. In other words, to deal with the AMR issue, the first step is to identify the causes and underlying contributing factors to this event in veterinary medicine and the disastrous conditions veterinarians face in Iran and the world. It is not possible to adequately approach what veterinarians go through and how they perceive the existing context only through quantitative research. A qualitative approach is needed to explore all aspects of this problem. Therefore, the present study employed this approach to explore the Iranian veterinarians’ perceptions of barriers to AMR prevention. We hope that the results generated from this study will help promote programs to curb slow down and the development of AMR in Iran and the world. The present findings can be used to make new social, economic, and even political decisions.

## Materials and methods

### Research design

This research was conducted with a qualitative approach and qualitative content analysis method from three cities of Iran: Kerman (with a large population of large and small livestock), Bandar Abbas (a fishing and aquaculture hub), and Tehran (with a large population of pets and industrial poultry). The present qualitative study used semi-structured interviews with veterinarians who had experience in treating and prescribing antibiotics or office work in veterinary medicine, or those with sufficient experience and knowledge of issues in veterinary diagnosis and treatment. The interviews were held face-to-face from May 3, 2021 to August 13, 2021. In this study, theme analysis was used, which is a common type of qualitative content analysis. It seeks a deep understanding of the complexity, details, and embedded context of a given phenomenon. In this type of analysis, interviews with individuals provide a better understanding and richer information about participants’ experiences and perspectives. This research approach allows for an in-depth and rich exploration of participants’ experiences.

The panel of experts offering advice on research questions and reviewing the transcripts for reliability consisted of one epidemiologist, three health education and health promotion specialists, and two veterinary professors collaborating with three organizations: (Hormozgan University of Medical Sciences, Veterinary Department of Kerman University and Iranian Veterinary Organization). The present participants were selected through maximum variation and snowball sampling.

### Participants

The research population consisted of veterinarians from three cities (Tehran, Kerman, and Bandar Abbas) in Iran, all dealing with a large population of livestock, poultry, and aquatic animals in 2021. The inclusion criteria were: veterinary work experience at least three years, the experience of therapeutic clinical work or working as a veterinary administrative and supervisory staff, willingness to participate (in the research), and ability to answer the questions. The exclusion criteria were unwillingness to participate and withdrawal from the interview. Purposive sampling was used with maximum variation (in terms of the province of work, age, sex, the field of work in a clinic or pharmacy, and affiliation with the public or private sector) at first steps and snowball sampling methods In the following. That means some veterinarians were concerned about expressing their opinions or reporting any illegal case they had dealt with. Therefore, they had to be selected through snowball sampling. After reaching the first participant and holding the interview, s/he was asked to suggest the next veterinarian who was aware of or was experienced in prescribing antibiotics against paraclinical rules. Therefore, each participant connected and introduced us to the next participant. Interviews were held in a public place at the interviewee’s convenience. In some cases, the interview was held in the clinic, and in others in the interviewee’s office. The data were collected and analyzed simultaneously. The interviews continued until the data were saturated (i.e. when no new information was obtained) and until all the extracted themes were sufficiently supported by the data. After the 17th interview, no new data were collected, but to be on the safe side, another interview was also conducted, and after the interview with the 18th participant, the sampling was stopped.

### Data collection

Guided questions and semi-structured interviews were used for data collection. The interviews were held face-to-face and video calls. When required, a trained research assistant conducted a qualitative interview to increase the accuracy and speed of data collection. The interview questions were derived from a review of the existing literature on AMR with a focus on the underlying causes and also the comments made by a panel of experts. At the beginning of the interview, the purpose of the study was revealed to the participants and they were assured of the confidentiality of the information they provided and the anonymity of their responses. The interviewees were ensured they could withdraw from the study upon their will. Then, an informed letter of consent was signed. The required permission was gained to record all the conversations. The main focus of the questions included:

1. What are the barriers to veterinarians’ prevention of increased AMR? Explain.
2. What do you know about the causes and precursors of AMR occurrence in Iran?
3. What are the determinants of prescribing and using antibiotics in veterinary medicine in Iran in your opinion? Explain.
4. What are the determinants of the increased AMR in your opinion as a veterinarian?

Based on the participants’ previous answers, more exploratory questions were asked and, as a result, we extracted the main reasons why veterinarians over-prescribed drugs and why antibiotics were overconsumed in the animal source food industry. The sample size was determined by the theoretical data saturation criterion. In other words, during the data collection, when we concluded that more interviews and observations could not add any new information and only led to repeated findings, we stopped the data collection. Therefore, 18 active veterinarians in clinical, medical, educational and administrative fields were interviewed in Tehran, Kerman and Hormozgan (provinces). Individual interviews lasted between 42 and 57 min.

### Data analysis

The process of data analysis was done using Granheim and Lundman method [29, 30] and with the help of MAXQDA-2010 software by the first and second authors of the article. The first and second authors listened to recorded interviews and transcribed them into a written format in Word 2017 software immediately after every interview and on the same day with the help of other research colleagues. In the second step, the text of the interviews was read by the researchers very carefully to get a general view of their text. In the third stage, all the texts of the interviews were read line by line and very carefully, and the initial codes were started.

In the fourth step, the researchers placed the codes that were similar in terms of meaning and concept and were placed in a category in a subcategory and determined the relationship between them. In the fifth step, the codes and categories were placed in the main categories, which were conceptually more comprehensive and abstract [31]. Finally, in a joint meeting, the entire process of data analysis was shared and conflicting opinions on the content of a topic were discussed by a research team with two qualitative health researchers and two veterinarians.

### Rigor

Guba and Lincoln evaluation criteria [32] were used to check the trustworthiness of the findings. To substantiate the validity of the findings, the researcher’s self-review technique was used in data collection and analysis as well as a peer check during which the codes were provided to two participants to resolve misunderstandings. To substantiate the reliability of findings, intra- and inter-rater reliability tests were used. To this aim, the recorded and transcribed conversations were given to several experts for review. After analyzing the data, they were re-analyzed by colleagues. The next step was documentation to test the accuracy and comprehensibility of the procedures, and the underlying mechanisms of errors.

### Ethical considerations

This research was approved by the Ethics Committee of Hormozgan University of Medical Sciences (IR.HUMS.REC.1400.207). In the interviews, the researcher, by introducing herself and also explaining the purposes of the study, tried to create an amicable atmosphere for the interview. The participants were also ensured of the confidentiality of the information they provided, the anonymity of recorded conversations, and also why they were selected. They consented to their voice being recorded. The participants were free to withdraw or leave the interview any time they requested.

## Results

The present study was conducted as interviews with 18 veterinarian participants in Tehran, Kerman, and Hormozgan provinces. Both sexes were included. There were 11 male and 7 female participants whose ages ranged between 27 and 58, with an average age of 42.5 years. The participants’ work experience ranged between 3 and 27 years, with an average of 15 years. The demographic information is summarized in Table 1.

**Table 1:** Research participants’ demographic information

Variable	Level	*N*.	%
Sex	Male	11	61
	Female	7	39
Age	< 30 years	5	28
	30–40 years	11	61
	> 40 years	2	11
Province of work	Tehran	4	22
	Kerman	9	50
	Hormozgan	5	28
Employment	private sector	11	61
	Public sector	7	39

The data analysis led to the extraction of 4 main categories and 44 subcategories (see Table 2), each examined separately.

**Table 2:** Subcategories and categories of AMR determinants

Categories	Sub-categories
Educational Factors	1. Unsystematic internship 2. Unadjusted curricula 3. Outdated education 4. Lack of specialized training courses 5. Low specialized study index 6. Lack of empowering educational system 7. Lacking cooperation of all medical sectors affiliated with the university
Administrative and Legal Factors	1.Problems with rules and regulations 2. Poor monitoring and adminisitration 3. Selling OTC drugs or those without laboratory-based approval 4. Inadequate advocacy 5. Lack of an interdisciplinary cooperative approach 6. Problems with the production and use of electronic health records (EHRs) 7. Slaughtering medically treated livestock 8. Non-compulsory training courses before issuing a license for animal husbandry 9. Lacking coordination between medical and veterinary organizations 10. Lacking attention to micro-industries and micro-breeders 11. Limited facilities in small towns
Client-related Factors	1. Quick response 2. Customer satisfaction 3. Low purchasing power 4. Social learning 5. Pharmaceutical determinants 6. Unawareness of antibiotic residues and abstinence interval 7. Drug replacement or early ceasure 8. Antibiotic use as a growth stimulant 9. Self-medication or arbitrary use of drugs 10. The unconventionality of the antibiogram test 11. Client’s preference for over-prescribing vets 12. Client’s lacking foresight 13. Rejection of paraclinical costs 14. Materialistic view
Veterinarian-related Factors	1. Lacking experience incorrect act of prescription 2. Prescription based on prior experience 3. Unconventionality of diagnostic tests among veterinarians 4. Being labeled as inexperienced if dependent on laboratory diagnosis 5. Fear of losing clients 6. Lacking foresight 7. Insignificance of AMR 8. Lack of self-efficacy in overcoming barriers 9. Diminishing ethical considerations 10. Competetive drug market 11. Job insecurity 12. Apparent issues with prescriptions

### Educational factors

The first determinant of the increased AMR deals with academic issues in university. Among the most important issues are those concerning students, clients’ lacking awareness and knowledge of AMR in animals, and its transmission to humans (from animal source foods).

#### Unsystematic internship

During the summer holidays of the final 2–3 academic years of veterinary students, they are required to take the internship. Yet, some participants complained about the unsystematic and inefficiency of this internship.

*“During our student days, we took up the internship, but we did not learn anything special at all”. (Participant #13)*

#### Unadjusted curricula

The majority of participants agreed that during their studies, only in the bacteriology course, they learn about AMR (only superficially) and that in the university curriculum, this subject was not adequately included.

*“All faculty members should teach something about AMR, not just the bacteriology professor. Also, do we not prescribe antibiotics once we diagnose viral diseases in clinical sciences and the like? If so, then why are we not taught what AMR actually is”? (Participant # 9)*

#### Outdated education

As the participants described, it was essential to teach new things about AMR and to develop strict, principled, written instructions on this subject. The participants recommended following effective and efficient exemplar instructions (in foreign countries) to strengthen the educational system not also at university but in food and drug administrative organizations. It is essential to update basic and clinical sciences curricula and add AMR to all courses, as most participants agreed.

*“We should keep up with the global community in this regard so that we can be fully aware of the new knowledge and instructions, and can create new instructions based on the preexisting ones”. (Participant #2)*

*”I think one thing that can definitely help is to see how successful projects in developed countries proceed. Let us follow their example”. (Participant #13)*

*”They still teach the way they did a hundred years ago. The subject matter should be changed. It seems as if discussing AMR does not matter at all”. (Participant #9)*

#### Lack of specialized training courses

A key determinant of AMR prevention was the need for useful and effective training courses for all those somehow affected or affected by the AMR, including vets, the health staff, medics, therapists, as well as the livestock and poultry breeders, and the like.

*“It is essential to hold relevant and useful training courses for ranchers, poultry farmers, pharmacists, as well as veterinarians, veterinary staff who perform inspection and monitoring work for others. So, everyone is expected to cooperate”. (Participant #2).*

*“Farmers should know that adhering to the (medical) interval helps decrease antibiotic concentration in the animal being treated. Thus, the farmer or breeder needs to postpone the slaughter time. Or he is advised not to consume animal source foods while they are being medically treated”. (Participant #7).*

#### Low specialized study index

As in many other sciences, gaining up-to-date knowledge requires studying the most recent research findings.

*“We should not only encourage those who influence drug resistance to study about this subject, but universities should also encourage professors to study more about the specialized topic. If a professor fails to have updated knowledge, s/he cannot teach students well. Thus, how can we expect the students to act efficiently in near future”? (Participant #5)*

#### Lack of empowering educational system

Some participants expressed concerns that the educational system did not adequately prepare students for accurate diagnosis and prescription in near future.

*“At university, nothing matters more than studying and getting good marks. The educational system does not actually prepare students for the work market. In other words, it does not simulate real conditions before students leave academic life and enter the work market”. (Participant #9)*

#### Lacking cooperation of all medical sectors affiliated with the university

In the present study, participants, all veterinary graduates or instructors, raised the question why discussing AMR was limited to the bacteriology course and not included in clinical and practical courses.

*“Why is medical resistance only limited to bacteriology? All other basic and clinical sciences sectors at university are talking about diagnosis and treatment, and are prescribing drugs. But, when they come to medical resistance, they only refer to bacteriologists and the bacteriology labs”. (Participant #9)*

### Administrative and legal factors

There are issues about the rules/regulations and policies on veterinarians’ practice and that of all people somehow concerned with animal source foods, which can add to the existing problems. Here are the categories and the relevant excerpts:

#### Problems with rules and regulations

As the participants pinpointed, there is a strong need for food safety rules and regulations especially in terms of AMR. Adherence to these rules and regulations should be closely monitored too.

*“Though there are rules, you can never be sure they are abided by fully. No one is afraid of not following the rules. Even I myself, who is doing clinical work, am not sure whether there is any prohibitory rule for this or not!” (Participant #8)*.

*“Breeders who administer drugs themselves or those who slaughter animals being medically treated should be fined or prosecuted because they threaten public health. But, in reality there is no way to stop them”. (Participant #5)*

#### Poor monitoring and administration

A number of participants acknowledged that even if there are rules and regulations, they are not fully observed. There has not been any efficient monitoring over how rules and regulations are followed. That is why rules have been ineffective.

“*All these are just instructions. In practice, there is no veterinary body monitoring how things are done. The rules are ineffective“(Participant #7)*.

#### Selling over-the-counter (OTC) drugs or those without laboratory-based approval

Selling all kinds of drugs, including antibiotics without prescription, without laboratory approval and freely in Iran has caused serious trouble.

“*You can get any medicine you want from any pharmacy at any time. Actually, the main customers of pharmacies are those who buy drugs arbitrarily”. (Participant# 11)*

”*In my opinion, pharmacies should not sell every kind of drug especially antibiotics unless they receive a laboratory approval for the antibiogram test. Likewise, a vet* should not prescribe antibiotics unless s/he receives the lab test result first”. (Participant #2)

”*In my opinion, the sale of medicine, especially antibiotics, should be subject to laboratory approval. That is, a person should not be allowed to buy medicine until the laboratory has determined the type of disease or at least the effective antibiotic, even if the vet has prescribed it”. (Participant #9)*

#### Inadequate advocacy

As some participants commented, gaining the full support of international, national and regional communities was a great issue.

“*We need the help of international and national organizations to solve this global issue. When a problem is global, the solution will definitely be achieved with the cooperation of international organizations”. (Participant #2)*

#### Lack of interdisciplinary cooperative approach

According to some participants, to achieve an optimal solution to this problem, all administrative, supervisory, diagnostic, and medical sectors should cooperate.

“*Solving this problem is not what only one organization can do. Universities should teach students in the right way; veterinary administrative organizations should do their job efficiently; the private sector (e.g., clinics and pharmacies) should obey the rules. Most importantly, there should be strict rules made and abided by with all sectors cooperating”. (Participant #4)*

”*Our clients should be aware of the importance of AMR and also aware of how the drugs are cycled among the environment, animals, and humans. The Environment and Veterinary Organization, public health and agriculture, and the like should all take serious actions. If one ring is missing from this chain, the whole chain is broken. All efforts will end up fruitless”. (Participant #6).*

#### Problems with the production and use of electronic health records (EHRs)

Developing systems such as the integrated prescription system and the use of EHRs can significantly help to prevent AMR occurrence.

“*If the EHR system was used, things would be better now. No pharmacy could then sell OTC drugs. Thus, no customer could buy antibiotics arbitrarily”. (Participant #14)*

#### Slaughtering medically treated livestock

According to some participants, a stock not responding to an antibiotic treatment does not need any lab test. Neither does it need any abstinence interval. It can easily gain slaughter permission even in emergency cases.

*“Here, an animal that is taking medicine and is not becoming well or is getting worse is sent for emergency slaughter. Is there any organization in charge here? Only if a buyer comes to know that an animal shows symptoms of a disease, he may buy it at a lower price”.*

*“Before the slaughter, the antibiotic residues are controlled in poultry but not in macro-livestock (e.g., cattle, sheep and goat)”. (Participant #12)*

#### Non-compulsory training courses before issuing a license for animal husbandry

Some participants insisted that the government should make it compulsory for applicants (for stockbreeding or husbandry) to complete AMR training courses before issuing a license for stock breeding. Here are some comments.

“*Certainly, people seeking for an establishment and operation license for livestock, poultry, fish ponds, and in short, any kind of livestock, must be obliged by the relevant governmental body to first pass a series of training courses and then get a license”. (Participant# 2)*

#### Lacking coordination between medical and veterinary organizations

Due to the lack of the required infrastructure in veterinary medicine, this organization needs to cooperate with the Ministry of Health (for service provision), and medical and laboratory sectors especially to perform laboratory tests.

“*We can say that great concern is that veterinary medicine and medical sciences are affiliated with two different ministries. The former is deprived of the facilities provided by the ministry of health. Even for simple antibiogram tests, we should visit veterinary labs provincial centers, or big cities”. (Participant #11)*

#### Lacking attention to micro-industries and micro-breeders

With the expansion and development of livestock, poultry, and aquaculture industries, the main attention has been focused on this group (of industries), and domestic and micro-breeders have been neglected.

“*If there are any rules and regulations, they are mostly about industries such as macro-level poultry breeding or husbandries. Yet, in practice, the national livestock is to a great extent bred by domestic and micro-level breeders that are largely neglected”. (Participant #3)*

#### Limited facilities in small towns

The lack of diagnostic facilities such as laboratories equipped with antibiogram testing for cases sent from veterinary clinics have caused serious problems for clients and veterinarians.

“*For a simple antibiogram test, we have to refer to the provincial center, and this is both time-consuming and costly. More importantly, most of our livestock population is in small towns, not in provincial centers”! (Participant #1)*

### Client-related factors

Another determinant of the increased AMR as perceived by Iranian veterinarians is the factors related to livestock/poultry breeding and animal owners (termed here as “clients”). The clients’ choices, decisions, and behaviors will have significant effects on increasing AMR.

***Quick response***: Among the reasons for an emergent antibiotic prescription without any diagnostic test are: concerns about high mortality rate if the drug is not used immediately, the breeder’s referral at the onset or peak of a disease spread, or substantial losses in the herd, or the referral rush to improve conditions.

“*Mostly, livestock farmers especially poultry farmers or any other breeder with a significant number of livestock, poultry, or aquatic animals, insist on getting a strong antibiotic immediately so that the mortality rate does not rise any further. They cannot even wait for the antibiogram test result. If we do not prescribe antibiotics for them, they go get it from somewhere else, and even if they go for the antibiogram test, they may not be patient enough to receive the test result and, thus, arbitrarily begin other antibiotics”. (Participant #6)*

#### Customer satisfaction

Some clients have used several specific drugs for years and found them effective. Thus, they have no faith in the lab diagnostic test result. Besides, some clinicians and especially vets are sometimes subject to too many demands, which can be tempting. They might occasionally be tempted to violate the existing rules and, upon a client’s persistence, they may neglect the protocols and easily give in.

“*For our clients, the drug manufacturing company even matters. Sometimes, they carry the former drug vial to show us and insist that the same drug be prescribed”. (Participant #3)*

”*Even when the required facilities were available, I faced too many suggestions. Some guys came to tell us to take it easy and let them get away with it (by granting or renewing their permit)”. (Participant #16)*

#### Low purchasing power

As there is no drug and treatment insurance for animals in veterinary medicine in Iran, the cost of treatment or the price of drugs was found as another determinant of antibiotic prescription, as mentioned by the present veterinarian participants.

“*Sometimes prescriptions are written out according to the customer’s affordance. Sometimes, customers ask us to prescribe something they can afford to buy. As there is no insurance coverage for veterinary medicine, the price matters, and it significantly affects the act of prescription”. (Participant #7)*

#### Social learning

An effective factor in antibiotic self-medication or arbitrary use of antibiotics is to learn about it. There are often others living in the same place (city or village) where the clients live, who used a certain drug and found it effective. Now the clients tend to follow their steps. Besides, self-medication cuts down on the diagnostic and therapeutic costs too.

*“A farmer might come to us and insist on buying the same drug that his neighbor has already bought. He does not consider that the diseases might be different. Overall, clients are more influenced by neighbors than us”!*

#### Pharmaceutical determinants

In some cases, what causes the clients to insist on our prescribing OTC drugs is the price and effectiveness of the drug (as perceived by the clients) and even the drug manufacturing company.

“*Some clients insist on buying a certain antibiotic because either they have already used and found it effective. Thus, they may ignore what the vet’s diagnosis is”. (Participant #11)*

#### Unawareness of antibiotic residues and abstinence interval

While using antibiotics, the livestock, poultry, and aquaculture breeders should be aware of the animal source food abstinence interval. But in reality, they are mostly unaware of that.

*“Many clients are not adequately aware of the abstinence interval after taking antibiotics, and this issue makes them send the animal products into the food cycle during the treatment period”. (Participant # 11)*

#### Drug replacement or early cessation

When seemingly the symptoms of the disease are gone, some breeders ignore the medical instructions and cease the drug sooner than they should.

*“For example, a drug should be taken for not shorter than a week. But when a client takes the drug for two days and feels the disease is gone, he stops administering the drug. He does not care about the medical resistance and how it occurs. He ignores them all”. (Participant # 5)*

*”A client may purchase an antibiotic (either prescribed or self-medicated) and begin the treatment. After one or two days, when there is no sign of recovery, he replaces the drug easily”. (Participant #8)*

#### Antibiotics use as a growth stimulant

Antibiotics have long been used as growth stimulants on a large scale by breeders of raw animal products in Iran.

“*In large breeding industries such as livestock and poultry farming, antibiotics are used as a growth stimulant, and this is very common”. (Participant #4)*

#### Self-medication or arbitrary use of drugs

As the participants mentioned, many clients take some therapeutic measures before visiting a veterinary diagnostic and medical center. They have already begun taking several antibiotics or have quit the treatment half in the way.

“*Sometimes a farmer arbitrarily buys and consumes several drugs before going to any veterinary, diagnostic or medical center”. (Participant #5)*

”*Some ranchers already take many antibiotics. When we ask them why they say they had it refrigerated since the last time they ever purchased and consumed the drug. They intended to use the remains of the drug and visit a clinic only if their self-medication did not prove effective”. (Participant #8)*

#### The unconventionality of the antibiogram test

As perceived by the present participants, antibiogram testing is a new thing that has not been yet received well by many clients. Not many participants welcome or even prioritize this test. They think doing this test is not compulsory and, thus, they do not feel obliged to take it at all.

*“Unless the customer is obliged to, he does not go for the test to a laboratory at all. Thus, it needs to be mandatory; yet in reality, it is not”! (Participant #15)*

”*When we tell a client that he should take a sample for an antibiogram test and wait until then, he is surprised. It seems as if he has never heard of such a thing. He wonders why none of his fellow breeders were already sent for such a test when they faced the same problem”. (Participant #18)*

”Nobody cares about AMR in the future. They laugh at us and wonder what the consequences are”. (Participant #2)

#### Clients’ preference for over-prescribing vets

As perceived by the present participants, any veterinarian or clinician who prescribes more drugs to treat animals is more popular.

“*If you do not prescribe any drug, the client prefers to go to another vet. He will not wait at all for you to tell him about the importance of drug resistance. Now every doctor who prescribes more drugs becomes more popular and he is perceived as a better doctor”. (Participant #18)*

#### Clients’ lacking foresight

Some participants acknowledged that the AMR problem is unthinkable in the future and far-fetched to many clients.

“*People do not really know what will happen in the future and people will suffer from drug resistance. No one can even imagine what will happen in the future. It is not tangible to them”. (Participant #2)*

#### Rejection of paraclinical costs

Some clients, as the participants’ accounts, revealed, do not bear costs higher than those of the visit, including the cost of a laboratory.

“*Our clients are mostly reluctant to pay much, especially when the cost of the treatment is higher than that of, for example, a domestic chicken that they bring here for treatment”. (Participant #18)*

#### Materialistic view

Many ranchers ignore many important things and are just concerned with more production and productivity, and gaining as much money as they can. So, they do things that are sometimes unethical and illegal but just cost-effective.

*“I don’t think it matters how you make money. You just need to be smart and know when to do what. For example, I know a guy who drugs his chickens the day before slaughter but keeps some of them apart for his own family’s use. He sends one of the undrugged chickens to the laboratory so that he gets a negative lab test result. This way, if there is any loss, it will happen to the undrugged chickens and not the whole poultry”. (Participant #9)*

*“Sometimes they breed a few chickens apart from others only to send them later on to the laboratory. The lab also cooperates with them and hides things in the actual report”. (Participant #13)*

### Veterinarian-related factors

In addition to the above-mentioned factors, veterinarians also sometimes cause an increase in AMR. Here we see how their characteristics affect their decisions on AMR development.

#### Inadequate job security

The current job market for veterinarians in Iran is not very prosperous and any factor that endangers the current position of activists in this field will fail.

*“If you cannot keep the customer satisfied with yourself in the job market right now and put extra costs on the customer, he will quickly go to another clinic and another vet”. (Participant #14)*

#### Lacking experience in the correct act of prescription

As perceived by the participants, many veterinarians who have just entered the work market lack any experience in prescribing drugs. Thus, they significantly account for the increased AMR.

“*As novice veterinarians do not have much experience in prescribing medicine, they prescribe several antibiotics together, with the hope that one of them works”. (Participant #13)*

#### Prescription based on prior experience

Prescribing drugs based solely on diagnostic experience is common practice in more experienced veterinarians.

*“ As soon as most colleagues see cases similar to what they have already faced and treated, they begin to write out the same prescription. It is generally well-established that certain drugs are always prescribed for respiratory infections, some for gastrointestinal infections, and so on”. (Participant #14)*

#### The unconventionality of diagnostic tests among veterinarians

Many veterinarians have diagnosed diseases and prescribed them mainly based on their own experience. Antibiogram testing is a new therapeutic measure that has not been welcomed warmly by vets.

*“There are very few vets who wait for the antibiogram test before writing out any prescription. Actually, antibiogram tests are still very uncommon”. (Participant #9)*

#### Being labeled as inexperienced if dependent on laboratory diagnosis

As our participants described, a veterinarian who does not make a diagnosis or give treatment immediately and independently (from lab tests) and hinders it until the paraclinical test results are labeled as inexperienced.

*“We have no problem sending the client to the lab, but unfortunately it seems as if we were unable to make a diagnosis ourselves and we were inexperienced and because of that we got help from the lab”. (Participant #12)*

#### Fear of losing clients

Some veterinarians acknowledged if they delayed the diagnosis to a later time (to receive the lab test result), they could easily lose many customers.

*“If you keep the client waiting or send him to a lab to fetch the test results, he will for sure prefer to visit another vet”. (Participant #5)*

#### Diminishing ethical values

Another determining factor raised by the participants was the need to have a working conscience and commitment to livestock/poultry breeders, laboratories, and those having contracts with labs. In other words, the vets should rely on the lab test results.

*“When I used to work on a poultry farm, I saw a separate hall for raising chickens with no antibiotics. The sample sent to the lab was taken from this hall. Or the chickens were slaughtered for the farmer’s own family. The other (drugged) chickens were sent to the slaughterhouse for public use”. (Participant #10)*

*“Some colleagues are not committed enough to their job and do not feel it on their conscience. Similarly, the test results coming from some labs are not reliable either. So, the negative antibiotic results we receive from them might be false”. (Participant #5)*

#### Lacking foresight

AMR is not familiar to many people in society. They do not adequately know what AMR is, which can affect their practice.

*“Veterinarians cannot even imagine how dangerous AMR can be to human health in the future. When they have no idea what AMR is and can be, how can we expect them to be worried about it”? (Participant #8)*

#### The insignificance of AMR

The AMR issue is not very important for some veterinarians in diagnosis, treatment, and monitoring.

*“Rarely does veterinarian care about drug resistance. I do not think it is even their last priority to consider”! (Participant #10)*

#### Lack of self-efficacy in overcoming barriers

A few interviewees admitted that they or some colleagues have a specific drug classification for most diseases according to which they act spontaneously. It means that they do not take different therapeutic measures when faced with different cases.

*“Some clinicians do not consider that everyone can have his disease. I mean, they treat all patients the same way and prescribe strong broad-spectrum antibiotics for 90% of cases”. (Participant #5)*

#### Competitive drug market

Many veterinarians are not required to sell drugs on a prescription, and selling without a prescription is a legal and common task. So active veterinarians in the field compete with each other for selling drugs and evidently for more income.

*“Everyone likes to open up a pharmacy because he can easily earn money with no trouble with diagnostic and surgical measures. It is much better if you can persuade customers to buy more”. (Participant #8)*

#### Apparent issues with prescriptions

The last subcategory of veterinarian-related factors was the appearance of prescriptions. The present prescriptions encourage vets to prescribe more drugs.

*“The size and shape of prescriptions are such that the vet is encouraged to prescribe more drugs. The prescriptions should be refined in shape to allow for one or two drugs only and no more”. (Participant #5)*

## Discussion

The present study aimed to explore the barriers faced by Irainian veterinarians against preventing Antimicrobial resistance. A few qualitative studies have been conducted on AMR, which dealt with the causes of progress and the obstacles faced by those involved in this problem, especially in the veterinary profession [33, 34]. The results showed that different educational, legal/administrative and veterinarian-related factors account for the increased AMR in Iranian society. The first category included factors related to the educational system, such as the lack of any specialized training course for veterinary students, those in charge of monitoring veterinary practice, veterinary departments, and ranchers struggling with educational problems who may all be implicated in increasing AMR. In Iran, various educational initiatives have been implemented, such as the publication of a book on rational prescription principles, academic papers, and reports from the National Committee on Prescribing and Rational Drug Use. Despite these efforts, there are numerous educational obstacles in veterinary colleges in Iran when it comes to instructing students on prescription fundamentals and the rational utilization of medications.

The required material has been also developed; training and retraining programs have been planned based on eclectic drug use criteria; workshops, conferences, and seminars have been held too. A prescription can simply be representative of a whole nation’s sociocultural values and medical conditions. Many studies have been conducted worldwide to improve rational drug prescription and consumption [35]. The effects of educational interventions on the improved prescription pattern have been reported in Iranians and other studies too [36, 37]. Continued training on rational drug prescription and pharmacy education has been recommended to doctors in the existing literature [38, 39].

In Zareh’s study, the most commonly prescribed drugs were injections and antibiotics. The research findings showed that, after the training, there was an increase in the rational prescriptions for most prescribed drugs [40]. As for teaching strategies, the WHO has published The Guide to Good Prescribing for medical students. This guidebook contains six rational steps that can significantly reduce the irrational prescription of drugs: 1- defining the patient’s problem 2- defining the goals of treatment 3- ensuring that the treatment is appropriate for the patient. 4 – initiating the therapeutic measure 5 - providing information, instructions and warnings (if any) 6 - monitoring and ceasing the treatment [41]. Outdated education was a sub-category found in this study. Different studies showed that dentists often, due to a lack of knowledge about the side effects of improper prescribing of antibiotics, tend to over-prescribe them [42, 43].

Veterinarians also are central to antimicrobial stewardship on farms, with their prescribing decisions significantly impacting AMR.A study on Canadian dairy cattle veterinarians’ revealed factors influencing their antimicrobial prescribing, attitudes towards reducing antimicrobial use, awareness of AMR, and perceived barriers to improving stewardship [44]. In addition educational resources have been developed to enhance veterinarians’ understanding of AMR and promote rational antimicrobial use. Online courses such as “Antimicrobial stewardship in veterinary practice” and “Farmed Animal Antimicrobial Stewardship Initiative” aim to educate veterinarians on responsible antimicrobial use [45, 46].

What we need is a high-quality time management element added to the existing curricula so that students can be well-equipped with whatever they need to act professionally. Excessive imitation of medical sciences in specialized courses can only lower the efficiency of a vet’s profession. Rather, there is a need for incorporating courses on different animal species both at the general practitioner’s level and the specialized doctorate degrees [47].

There is also the issue of time management in the curriculum. Decreasing the quantity of content and increasing the quality (by adding more useful content) can better reform the veterinary curriculum. Goal-setting in veterinary sciences has already been revolutionized, and veterinary universities cannot ignore it. Thus, it is essential to consider the present and future needs in defining the required specialties to handle the existing national health issues, each of which can impose a loss of millions of dollars nationally. For many years, curricula have been developed in the European Union to achieve the necessary specializations by the existing needs, at least in the cattle breeding industry [48]. A deficient educational system is one factor that increases the overuse of antibiotics. Therefore, it is necessary to take basic measures based on the macro-planning of students’ knowledge and increase the quality of internships. In a study by Wushouer et al. in China, it was observed that an increasing awareness was followed by a decreasing rate of antibiotic administration [49]. Therefore, it is necessary to increase knowledge through a different approach in the educational system. Most experts believe that education in medical sciences should follow a different approach than other fields of study because knowledge construction in these fields of study (i.e., medicine, veterinary medicine) affects the content that students receive and the experiences they gain [50].

Failure to hold training courses for producers of raw animal products and unsystematic student internships can significantly lower the quality of education. Raising the study index in AMR and modeling on successful examples can be considered in curriculum design. Moreover, all departments of the veterinary faculties should cooperate and the heavy burden of teaching AMR should be removed from the bacteriology department only, and be shared by all basic sciences and clinical courses. Only then can we hope to see improved practice in students’ learning experiences and professional life in the near future.

The present findings showed that currently in our country, the educational system needs to be seriously reformed by appropriate training programs and pre-employment awareness-raising programs for veterinarians and ranchers [51]. People working in this field should be more empowered, better aware, and skilled enough at a correct diagnosis or proper functioning [52]. Only then we can hope that their self-efficacy is increased and they can learn to act more responsibly. These can help to prevent the occurrence of AMR and to begin to resolve it rather than worsening the issue.

The second category of the determinants of increased AMR was administrative and legal factors. Problems with the law, monitoring, and selling OTC drugs are important issues that can increase the costs of treatment too. This finding is consistent with several studies. For example, it is estimated that about 100,000 people in the United States die every year from the adverse effects of drugs [53]. In the United Kingdom, problems in 11% of prescriptions cost over € 400 million in loss, and about 16% of these problems harm patients [54]. Most of these errors are preventable, including drugs prescribed heedless of contraindications, those taken incorrectly, or those not having been properly monitored. The WHO, along with other relevant international organizations, proposed certain criteria to evaluate the quality of prescriptions to prevent the occurrence of problems and lower treatment costs [55, 56]. A useful way of evaluating the prescription pattern in a country is to evaluate the doctors’ prescriptions. A simple prescription can represent the current state of medical education in a country, how laws and regulations affect the medical community, socio-cultural beliefs, and the medical condition [36]. Based on WHO guidelines use of medically important antimicrobials in food-producing animals, any level of restriction in antibiotic prescription should be considered, including a complete cessation of the use of one or more antibiotics. Examples of restrictions that WHO considered are: any prohibition on the use of antibiotics, such as but not limited to the prohibited use for specific indications (e.g., for prophylaxis of disease or growth promotion), the requirement of a prescription by a veterinarian for the use of antibiotics, voluntary restrictions on farms or organic interventions [55]. Drugs that need confirmation from a specially qualified person or organization should not be sold over the counter. Prescribed drugs are regulated by the US Food and Drug Administration (FDA). Having a federal license with a medical leaflet is a prerequisite for the packing of any drug. A medical leaflet usually consists of four parts: indications, contraindications, warnings, and dosage [57]. He who writes out a prescription decides who can consume the drug. A pharmacist can buy drugs, but he should sell them only to those authorized by a legally qualified person. Thus, a prescribed drug has 3 parts [58]: (1) The doctor’s prescription, (2) The pharmacist’s written prescription while delivering the drug, and (3) the drug package with a label on it. That is why officials are expected to always think about formulating new and public policies to implement correct and new strategies for the use of antibiotics [59]. Educational and political interventions, establishing and implementing laws regarding AMR stewardship may be effective and acceptable either before or during the livestock and poultry breeding programs, even for pet owners [60]. Success in the coordinated implementation of related laws is not possible without the advocacy of various stakeholders, including policymakers, veterinarians and ranchers, pet owners, public sector employees, farmers, and consumers [51]. It seems that the use of effective legislation, contractual requirements, professional obligations and the distribution of suitable facilities in more distant areas makes the implementation of this plan possible [21, 22].

The third category was the client-related factors. Quick response and arbitrary drug use were among the sub-categories. With the expansion of public access to the internet system, people may want to refer less to vets and, instead, self-medicate or they may expect a quick response and begin to use antibiotics. In their research, Hofmeister et al. investigated veterinary visitors and found the internet connection speed as the third most important source of retrieving pet health information after GPs and specialized vets and before family and friends and other mass media [61]. Kogan et al. maintained that internet-based sources are considered an extra source of information about pet health for pet owners besides visiting vets for consultation [62]. Volk et al. reckoned that the internet and online health information could replace veterinarians and lead to fewer pet owners visiting veterinary clinics [63]. Thus, since some clients do not want to pay the visit and para clinic fees, by searching on the Internet and cyberspace, or based on their previous experience or else, they prescribe and take antibiotics arbitrarily before any visit to vets. If they do not find some proper treatment, they try other antibiotics, which leads to the problem of changing or stopping the antibiotics early before the end of the treatment period.

Some other poultry or aquatic breeders who have farms of several thousand pieces are very worried about the loss of their livestock, poultry and the aquatic population at the beginning of the disease. Since there may be a large population of their herd while waiting for the antibiogram test, they prefer to use a broad-spectrum and preferably cheaper antibiotic (for large-scale use for a large herd) to begin with and prevent their economic loss to a large extent [60]. Therefore, both in the producers and breeders of animal-origin food and in the owners of pets, the customer’s demand needs quick response and the customer’s demand should be prioritized [22].

In addition, a person who once used a broad-spectrum antibiotic without a prescription and got a response, suggests that to his/her colleagues or other breeders, and by promoting social learning, this behavior promotes the progress of antimicrobial resistance. In addition, many of these people are unaware of antibiotic residues and abstinence intervals, and currently do not feel threatened about the future of antimicrobial resistance. When they go to the vet, they prefer to go to a vet who prescribes some antibiotics to return home without any drug prescribed [33].

Another subcategory extracted from the present findings was the use of antibiotics as growth stimulants by poultry farmers. The use of antibiotics, both as a treatment in humans and as a therapeutic measure or growth stimulant in animals, has a great effect on the microbial flora of the intestine and also induces resistant strains in these animals [64]. When used as a growth stimulant, antibiotics can have adverse effects on humans and animals [65].

The fourth category was the veterinarian-related factors. The lack of an inter-sectoral approach was one subcategory extracted from the findings. Foreign studies mentioned a multi-sectoral approach and knowledge sharing in educational environments [66]. There seems to be a need for all institutions to have the required knowledge about the use of antibiotics through shared efforts between universities, the government, and the various professions. One subcategory was the insignificance of antimicrobial resistance to veterinarians. Antibiotic-containing products have harmful effects and there is a significant increase in the resistance of different types of infectious bacteria [67] besides the important role that antibiotic-containing animal products play in this process. Thus, global efforts are needed to reduce antibiotic use and attempt to control it. More control is needed over veterinary drugs and their use in livestock and poultry farms [68].

In line with the qualitative study in the UK, this study showed various behavioral and contextual factors involved in the participants’ beliefs about AMR stewardship and their responsibilities in the right direction [33]. One of these issues is the lack of experience in writing correct prescriptions among novice vets who prescribe several antibiotics at the same time in the hope that at least one works. This finding is in line with some studies that acknowledged that, when uncertain, most new clinicians tend to over-treat with antimicrobial drugs instead of refraining from treatment [1, 69–71]. They prescribe several antibiotics in the hope that one works. The other extreme case is also possible when experienced veterinarians prescribe drugs based on their long-held experience. These clinicians have more faith in a series of antibiotics. On the other hand, the diagnosis of the disease and the prescription are dependent n each other. When they are told about the laboratory evidence, they react as if their credibility has been damaged. Therefore, they provide waves of unprincipled recommendations and increase antimicrobial resistance. If a veterinarian intends to prescribe antibiotics based on the principles and guidelines, s/he will face other problems, including the fear of losing clients because, as mentioned earlier, if the prescription is not in accordance with the client’s wishes or the urgency of responding to it, the client will prefer to go to another vet, and this issue will endanger the job security even more.

Another sub-category is lacking self-efficacy in dealing with different visits.In other words, the approach of veterinarians to prescribing antibiotics is to a great extent pre-established and classified. For example, oxytetracycline is the preferred antibiotic for most respiratory diseases. Any cause of disease that requires more attention to the self-efficacy of veterinarians and clinicians can be improved by training methods and participation in appropriate courses. Diminishing moral values ​​becomes important in cases where full-time monitoring of antibiotic residues in animal products and their transfer to society and the environment is not possible, and where the government and regulatory agencies fail due to poor enforcement of laws. The regulatory forces cannot monitor and take care of the veterinary private sector employees and breeders. We can only hope that the vets will feel committed enough in their acts of diagnosis and prescription and the resultant effect on antimicrobial resistance. In the end, it is possible to recommend the modification of the appearance of prescriptions as a solution, because most of the headers of the veterinarians’ prescriptions in Iran are designed in a large way, which encourages the person to fill most of the prescription with writing the unnecessary drugs, so maybe it is recommended to design and implement a single protocol in limiting the written space of the prescriptions, we can take a step in reducing the obstacles facing the control of antimicrobial resistance.

### Limitations, strengths and future directions

There were certain limitations in this study. As the interviews were face-to-face, participants might have been tempted to provide socially acceptable answers. Also, some veterinarians showed concerns about the illegal cases they were aware of and reported. So, they were selected through snowball sampling. In addition, selecting interviewees with work experience and an adequate understanding of the relevant problems and interviewing them in a private place were somehow difficult.

As in other qualitative studies, researchers’ beliefs may have influenced the study procedure from conceptualization to interaction with participants and data interpretation [72].There were chances that the interviewees’ comments did not cover all factors possibly because of the limited sample size. Sampling in qualitative studies continues until the saturation happens. Thus, in this study also the interviews continued until the data were saturated (i.e., when no new information was obtained) and until all the extracted themes were sufficiently supported by the data. No formula was included.

It is possible that besides the factors mentioned by the present participants, other experiences are gained in other parts of the country that cannot be subsumed under the present categories.

Despite the potential limitations, the present study has several strengths. The first is the sampling method with maximum variation (in terms of the province of work, age, sex, and ​​work in the clinic or drug supply or employed in public and private sectors). The next strength is that during the interviews, some participants were dissatisfied with the current conditions, and this study provided an opportunity for them to find solutions. Moreover, there has been extensive research on AMR, but the vast majority of them are quantitative. Few have explored AMR determinants in society. The present study goes beyond the laboratory work, and with the One Health approach, using numerous interviews, it gains a deep understanding of work experience, and comprehensive and valid data to solve the AMR issue. The authors of this study intend to use the data from this study or at least part of the data for future educational interventions. A focus on the categories extracted from these studies helps to plan effective multidimensional interventions. This study can also guide future lines of research.

## Conclusion

The results showed that AMR in veterinary medicine induced by veterinarians active in the clinical field occurs under the influence of different factors. To increase AMR stewardship, in the first step, the barriers facing all people involved should be deeply studied and identified. Appropriate plans and policies should be made to deal with the underlying factors. Educational, administrative and legal, client-related factors, and veterinarian-related factors should be considered as the determinants of the increased AMR. It is essential to reform the education system and strengthhen the interdisciplinary relationships, especially among universities and between the university and regulatory organizations. Removing the barriers these people face and reducing the consequent trouble can make the widespread emergence of AMR more evident. Its adverse effects on society will become a crisis which increases the causes of mortality due to the resistance produced to the antibiotics prescribed to patients.

## Data Availability

The original contributions presented in the study are included in the article/supplementary materials, further inquiries can be directed to the corresponding authors.

## Abbreviations

AMR: Antimicrobial Resistance
OTC: over-the-counter
